# Editorial: Novel insights into the regulatory role of sugar and amino acids signaling in plant-microbe interactions

**DOI:** 10.3389/fpls.2023.1261186

**Published:** 2024-01-04

**Authors:** Yuan Hu Xuan, Yiming Wang, Li Gao, Xiangchao Gan

**Affiliations:** ^1^ College of Plant Protection, Shenyang Agricultural University, Shenyang, China; ^2^ College of Plant Protection, Nanjing Agricultural University, Nanjing, China; ^3^ Institute of Plant Protection, Chinese Academy of Agricultural Science, Beijing, China; ^4^ Department of Comparative Development and Genetics, Max Planck Institute for Plant Breeding Research, Cologne, Germany

**Keywords:** plant, microbe, interaction, sugar, amino acids

Food security and safety are important issues due to the expanding human population. As sessile organisms, plants have evolved sophisticated mechanisms to cope with pathogens. Plant growth and development require the acquisition and transport of nutrients that mediate cellular signaling in the plant and activate the expression of growth-promoting and/or anti-pathogen genes. Nutrients, including sugars and amino acids, are necessary for high-yield crop production but are also tightly associated with plant-microbe interactions. Microbes utilize several strategies to adapt to plants, including enhanced root cell surface for absorbing nutrients, competing for environmental nutrients, hijacking plant nutrients, and altering cellular nutrient transport and signaling. These beneficial or harmful effects lead to a shift in the plant microbiome. Therefore, analyzing the role that nutrients play in plant defense is of critical importance to boost the efficacy of fertilization.

Fusarium head blight (FHB) severely threatens wheat quality and production. Zhao et al. analyzed metabolites of the resistant genotype Sumai 3 and the susceptible genotype Shannong 20, after *Fusarium graminearum* inoculation. The results showed that some of the amino acid contents changed significantly in different cultivars, and that the exogenous application of proline (Pro) and alanine (Ala) increased wheat resistance to FHB, while cysteine (Cys) aggravated the susceptibility, suggesting tight association of amino acid metabolism and resistance in wheat. *Fusarium oxysporum* is a main causative agent of tobacco root rot, severely affecting tobacco growth. The examination of the virulence of 200 F*. oxysporum* strains as well as the identification of expression patterns positively correlated genes with virulence levels and demonstrated that ATP synthetase genes are important for *F. oxysporum* virulence by suppressing tobacco expression levels of *sugar will eventually be exported transporters* (*SWEETs*) [Gai et al.]. The root-knot nematode *Meloidogyne incognita* infection significantly changed the expression levels of *SWEETs* in Arabidopsis. Histological and genetic analyses indicated that *M. incognita* infection induced the expression of *AtSWEET1* specifically at the galls, and mutation of Atsweet1 dramatically promoted plant resistance to *M. incognita* [Zhou et al.], suggesting that SWEET sugar transporters might be the targets of different type of pathogens for disease occurrence.

Common smut, caused by *Ustilago maydis* (DC.) Corda, is a destructive fungal worldwide disease in maize. To explore the defense mechanism of maize to *U. mydis*, transcriptome and subsequent genetic analyses were performed. The results indicated that hexose metabolism is significantly altered by infection of *U. mydis*. Further, the expression level of the *Galactinol-sucrose galactosyltransferase* (*GSG*) gene is sensitive to *U. mydis* infection, and *GSG* negatively regulates maize resistance to common smut, implying a connection between hexose metabolism and common smut disease [Zou et al.]. Reactive oxygen species (ROS) accumulation is a key signal of plant defense. Zhang et al. identified a chloroplast peptide chain release factor mutant *dig8*, in which abnormal thylakoid stack formation and chloroplast dysfunction were observed. Moreover, the *dig8* mutant caused increased ROS activity, leading to callose deposition; however, a local sugar supplement partially alleviated the *dig8* mutant phenotype, suggesting a potential link between sugar synthesis and ROS generation in plants.

Elucidation of the molecular basis related to amino acids and sugar metabolism and transport could help us to improve strategies for efficient plant protection. The excellent studies mentioned here demonstrate the importance of the research community in understanding and explaining novel insights on the regulatory role of sugar and amino acids signaling in plant-microbe interactions. In the future, the application of certain amino acids and Crispr-cas9-mediated genome-editing of susceptible genes involved in amino acids and sugar metabolism or transport would greatly improve plant protection against pathogens.

## Author contributions

YX: Writing – original draft, Investigation, Supervision. YW: Writing – review & editing. LG: Writing – review & editing. XG: Writing – review & editing.

